# Influence of Formate Concentration on the Rheology and Thermal Degradation of Xanthan Gum

**DOI:** 10.3390/polym13193378

**Published:** 2021-09-30

**Authors:** María José Martín-Alfonso, Javier Mauricio Loaiza, Clara Delgado-Sánchez, Francisco José Martínez-Boza

**Affiliations:** Centro de Investigación en Tecnología de Procesos y Productos Químicos (Pro2TecS), ETSI, Universidad de Huelva, 21071 Huelva, Spain; javiermauricio.loiza@diq.uhu.es (J.M.L.); clara.delgado@diq.uhu.es (C.D.-S.)

**Keywords:** xanthan gum, potassium formate, rheological behavior, high-temperature aging

## Abstract

Xanthan gum solutions have gained increasing interest for their use as environmentally friendly chemicals in the oil industry. Xanthan is compatible with most concentrate brines used for controlling formation damage and fluid loss. Particularly, formate brines reinforce the ordered structure of the biopolymer in solution, gel strength, and the specific gravity of the resulting fluid. In this paper, we studied the effect of thermal aging on the rheological behavior of xanthan solutions as a function of the concentration in potassium formate. Ionic strength below a threshold concentration does not prevent the degradation of the structure of xanthan after being submitted to aging at 165 °C. Aged solutions show an important loss of strength in their mechanical properties, lower pH, and higher content in furfural and hydroxymethylfurfural. Highly concentrated formate brines are necessary to maintain the strength of the rheological properties after exposure to high-temperature environments.

## 1. Introduction

Biopolymers have been proven to be an interesting alternative for the development of sustainable and environmentally friendly fluids in many industrial activities. Polysaccharides, such as xanthan gum (XG), in brine solutions are commonly used in oilfields as rheology controllers. Its pseudoplastic characteristics and high resistance to shear degradation [1,2] cover the desired requirement of high viscosity at a low shear rate, which is necessary for maintaining drill cutting in suspension when the flow is stopped, avoiding settling to the bottom hole. At the same time, these characteristics cover the desired requirement of low viscosity at a high shear rate, which facilitates pumping circulation [3,4,5,6,7]. Particularly, formate salts have been used successfully since the late 1990s as completion fluids due to their high solubility in water and the ease with which one can prepare formulations with the required density to compensate the formation pressure [8,9].

The rheological versatility of XG in solution is due to the development of a time-dependent structure with a well-known order-disorder transition that leads to non-Newtonian behavior, which depends on concentration, temperature, pH, ionic strength, pyruvate and acetate content, etc. [10,11]. The rheological behavior of XG has been characterized as weak gel-like in linear oscillatory shear [12,13,14,15] and pseudoplastic in viscous flow [16]. This behavior turns it into a Newtonian liquid at higher temperatures with the disruption of the ordered structure above the conformational thermal transition [17].

The presence of salts in XG solutions not only improves the strength of the rheological behavior but also enhances the thermal resistance to degradations at high temperatures [8]. High ionic strength protects the helical structure, favoring the ordered state and increasing the order-disorder transition temperature, and consequently preventing XG chains from chemical attack [18], mainly in highly concentrated formate brines [9]. Despite the screening protection of the structure, degradation always takes place with thermal aging [7,19,20,21]. Degradation of the ordered xanthan heavily influences its rheological behavior, turning its gel-like properties to liquid, and consequently, there is the challenge of extending the performance window for engineering oilfield applications [22,23,24,25,26].

To go further into the role that formate salts play in the stabilization of xanthan solutions at high temperatures, in previous works, the rheological behavior of XG as a function of the XG concentration in concentrate brines has been studied, concluding that potassium formate enlarges the range of temperatures at which the solution behaves as a weak gel [9], enhancing its resistance to thermal degradation and improving the conservation of the pseudoplasticity [20].

In this paper, we focus on the effect that ionic strength has due to potassium formate on the rheological behavior of XG solutions, exploring the change in pH and the products of degradation, before and after being submitted to thermal aging. It was hypothesized that the screening effect and association promoted due to the high concentration in potassium formate would protect the ordered structure of XG in solution against the chemical attack at high temperature, avoiding the degradation of the non-Newtonian behavior.

## 2. Materials and Methods

### 2.1. Sample Preparation

A native xanthan solution, 2.0 wt%, was prepared by adding xanthan powder (Mw ~·10^6^ g/mol; Guinama S.L.,Valencia, Spain) without purification to distilled water; 0.01 wt% sodium azide (Sigma-Aldrich Co., Steinheim am Albuch, Germany) was added to the solution as a preservative. The sample was maintained at rest to fully hydrate for 24 h, after which the solution was stirred at room temperature in a Silverson mixer (Silverson Machines Ltd, Chesham, UK) (600 and 2000 rpm for 15 min, respectively). This stock solution was stored at room temperature.

Solutions of 100 mL of XG in potassium formate were made by taking 25 g of the native xanthan stock solution at 2.0 wt%, dissolving the corresponding quantities of reagent grade potassium formate (Sigma-Aldrich Co., Steinheim am Albuch, Germany), and adding distilled water to complete the volume of 100 mL. A magnetic stirrer (C-MAG HS 7, IKA-Werke GmbH, Staufen, Germany), at 500 rpm, was used to homogenize the solution.

The densities of the solutions were measured at 30 °C, taking the clear liquid after separating the xanthan gel by centrifugation at 6000 rpm (MAGNUS 22, Ortoalresa, Daganzo, Spain), using an Anton Paar DMA 5000 densimeter.

### 2.2. Rheological Measurements

The rheological characterization was carried out using a controlled stress rheometer Physica MCR-301 (Anton Paar, Seiersberg, Austria), equipped with coaxial cylinder geometry CC27 (26.266 mm inner diameter, 28.92 mm outer diameter, and 40.032 mm length), that was used for all measurements, at the temperature of 30 °C, which was controlled by a Peltier system C-PTD200.

The linear viscoelasticity region was determined by performing stress sweeps on each sample at a frequency of 1 rad/s, at the temperature of 30 °C. Frequency sweeps, from 0.1 to 100 rad/s, were carried out at shear stress inside the linear viscoelasticity region. At least two replicates of each test were undertaken. The standard deviation among replicates was within ±5%.

Steady-state viscosity curves were collected at 30 °C, increasing the shear rate (upward curve) and then decreasing the shear rate, with the data being collected over a period of 60 s at each shear rate and using the continuous shear rate mode option of the rheometer. The two sets of data were very similar (within 5% of each other). As such, only the downward flow curves are presented here.

### 2.3. Thermal Aging and Degradation

Samples for aging were placed in high-pressure containers (100 mL) made of 316L stainless steel and were pressurized with 50 bar of nitrogen at room temperature. The samples were statically aged at 165 °C for 16 h in a convection oven (Heraterm, Thermofisher, Waltham, MA, USA). Pressure and temperature were recorded during the whole aging period with the help of a pressure transmitter Sensotec DMP 331, a thermocouple type K, and a DAC device inet-555 (Omega Engineering, Norwalk, CT, USA). After aging, samples were cooled to room temperature and stored at 4 °C for the measurement of the rheology, density, and furfural/ hydroxymethylfurfural (F+HMF) content.

F+HMF was quantified before and after aging by ultraviolet-visible (UV-vis) spectroscopy, using a GENESYS 10 UV-Vis Spectrophotometer (Thermo Fisher Scientific, Waltham, MA, USA) equipped with quartz cuvettes of 1 cm light path. Samples were centrifuged (MAGNUS 22, Ortoalresa, Daganzo, Spain) to remove precipitates, and the supernatant was diluted and analyzed. A sweep for wavelengths from 200 to 800 nm was performed (results not shown) and the maximum peak was detected in the range 272–282 nm. A wavelength of 277 nm was selected for testing in accordance with the work by Heggset et al. [26]. The calibration curve for F+HMF was constructed from solutions of Sigma-Aldrich standard (Lot 185914/Lot W501808) containing a 1–20 ppm concentration of F+HMF fitted to the equation:

(1) $$A=0.149\left[F+\mathrm{HMF}\right]+0.066\;\;\;\;\;\;\left(R^{2}=0.999\right)$$

where A is the absorbance at 277 nm, which ranged from 0.122 to 1.037, and [F+HMF] is the furfural and hydroxymethylfurfural concentration in ppm. The samples were diluted 1/50 for spectrophotometric measurement. Each experimental value was the average of five results. Deviations from the respective means were all less than 1%.

## 3. Results and Discussion

### 3.1. Viscoelastic Behavior

**Figure 1:**
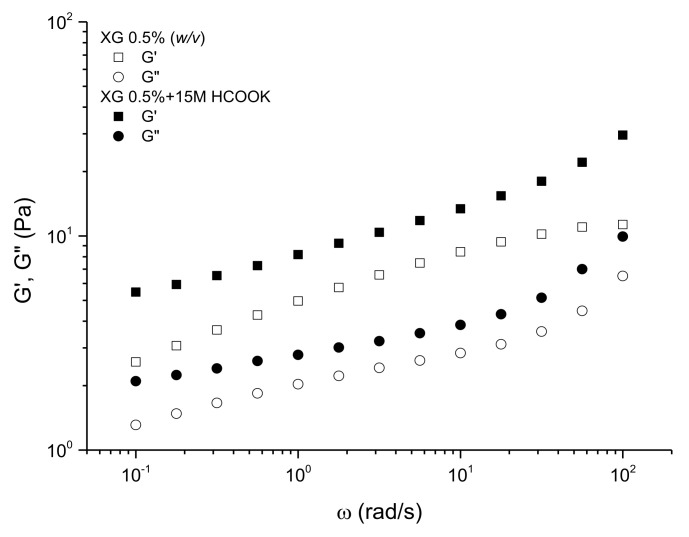
Storage and loss moduli versus the frequency for fresh XG solutions at 0.5% (*w/v*).

Figure 1 shows both the storage and loss moduli versus frequency for fresh xanthan solutions in distilled water and in 15.00 M of potassium formate (HCOOK), at the temperature of 30 °C, well below the conformational thermal transition of XG in solution [15]. As can be observed, for both solutions, the values of the storage modulus are higher than those of the loss modulus at the same frequency. In addition, both storage and loss moduli show a weak dependence on frequency. This is more apparent for the solution in formate brine, which shows a lower slope in the evolution of moduli with frequency at low and intermediate frequencies. These observations demonstrate that these solutions behave as weak gels. Gel-like behavior is typical for XG solutions, at moderate concentrations, where the intermolecular associations control the response to linear deformations, as has been previously reported [12]. The presence of a high concentration of salts compacts the ordered structure of XG in solution, enhancing the molecular interactions and the association by hydrogen bonding [27]. This effect increases the strength of the viscoelastic response of the structure, leading to both the increase in the values of the storage and loss moduli and the decrease of the loss tangent (tan δ = G’’/G’) [28]. Similar behavior has been described for concentrate XG solutions in chloride and formate brine [9] and in sodium and calcium chloride [21].

**Figure 2:**
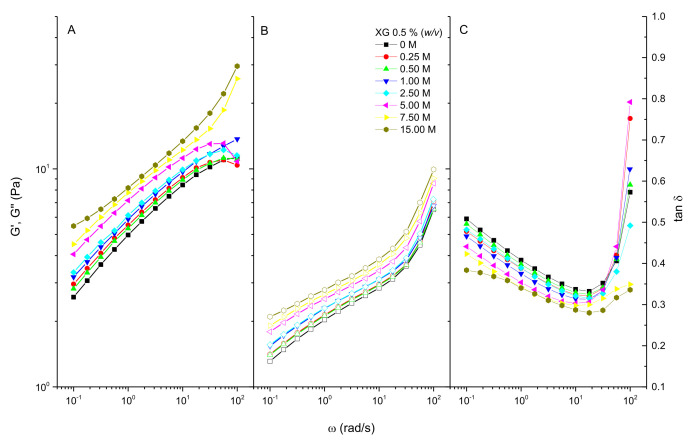
Evolution of the viscoelastic properties with the potassium formate concentration for fresh XG solutions at 0.5% (*w/v*) in HCOOK. (**A**) Storage modulus. (**B**) Loss modulus. (**C**) Loss tangent.

Figure 2 shows the evolution of the viscoelastic moduli as a function of the potassium formate concentration for fresh XG solutions. As can be observed in Figure 2, the values of both the storage (Figure 2A) and loss modulus (Figure 2B) increase when the molar concentration of formate increases in the solution. At the same time, the loss tangent decreases with both the frequency and concentration, up to a minimum value of around 20 rad/s. After that, tan δ values increase suddenly (Figure 2C).

**Figure 3:**
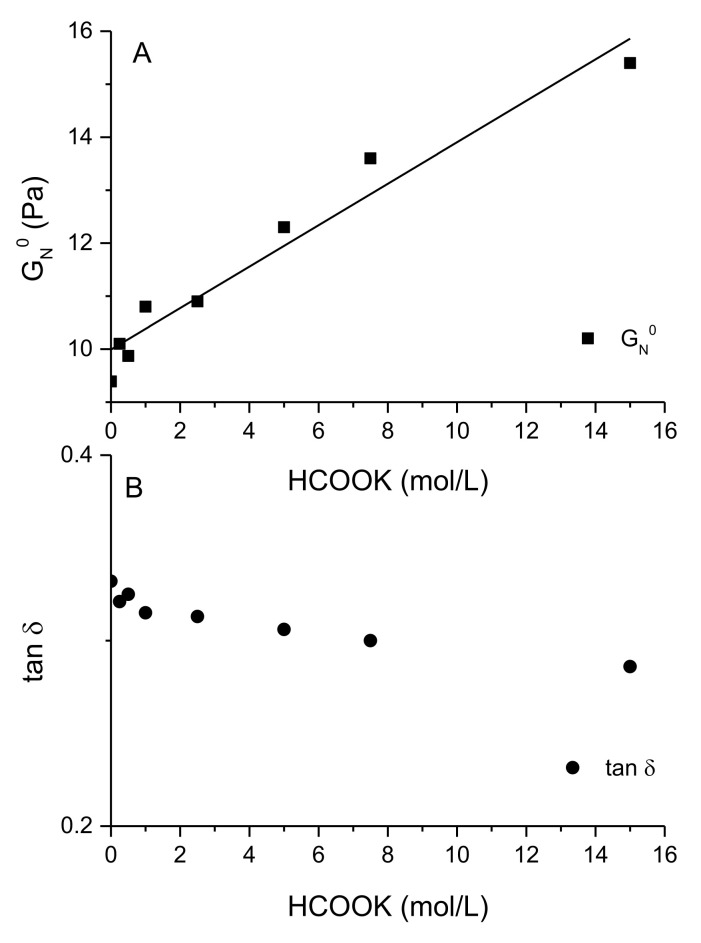
Evolution of the plateau modulus (**A**) and the minimum of tan δ (**B**) with potassium formate concentrations for fresh XG solutions at 0.5% (*w/v*).

To quantitatively evaluate the effect of potassium formate on the viscoelasticity of these solutions, a plateau modulus (G_N_^0^) is defined as the value of the storage modulus at the frequency of the minimum value of the loss tangent. Figure 3A,B display the values of both the plateau modulus and loss tangent at the frequency where the minimum in the loss tangent appears, as a function of the formate concentration, respectively. As can be seen in Figure 3A, G_N_^0^ increases nearly linear (R^2^ = 0.94) with the formate concentration from 9.4 Pa for the native solution to 15.4 Pa for the concentrated solution, respectively. Moreover, a slight decrease of the loss tangent is observed when increasing the formate concentration, from a value of 0.33 to 0.28 for native and concentrate solutions, respectively. Wyatt et al. [17,27] studied the effect of ionic strength on the rheology of XG solutions over a wide range of XG concentrations, observing that for moderate XG concentrations (above 2000 ppm), ionic strength plays different roles depending on the ionic concentration scale. In contrast, for this XG solution studied at a moderate concentration (0.5% *w/v*), 5000 ppm), a clear increase in rheology was observed with the concentration of formate over the whole range of ionic strength explored. A screening effect and Colombian repulsion would explain the changes observed in rheology with respect to native XG solutions in the low region of salt concentration [29]. Nevertheless, for higher ionic strengths (above, 1.00 M), the screening effect would reinforce interactions due to hydrogen bonding that would be responsible for the observed slight increase in the viscoelasticity and viscous properties, in agreement with the hypothesis of Wyatt et al. [27] of strengthening the hydrogen bonding by screening of the charge in concentrate brines.

### 3.2. Flow Behavior

At room temperature, below the conformational transition, XG in solution adopts a partial self-associated structure that is very sensitive to shear, showing a shear-thinning behavior due to the orientation of the polymer chains in the direction of flow as it has been previously reported [12,15,30]. Depending on the concentration and the experimental shear-rate windows, XG may show a tendency to reach a zero-shear limiting viscosity in the low shear-rate region; a power-law drop in viscosity at intermediate shear-rates that, in some cases, is followed by a trend towards an infinite shear-rate-limiting viscosity in the high shear-rate region. This shear-thinning behavior has been modelled by using different approaches, such as the power-law model [31], the Hershel-Bulkley model [15], the Carreau model [2], and the Cross model [9,11,20].

In this case, the parameters that characterize the shear-thinning behavior have been obtained from the Cross model Equation (2) [32] (viscosity η, shear rate $\dot{\gamma }$, limiting viscosities at low and high shear-rate η_o_ and η_∞_, respectively, the flow index m, and the consistency index k. Values are shown in Table 1:

(2) $$\eta =\eta_{\infty }+\frac{\eta_{o}-\eta_{\infty }}{1+{\left(k\cdot \dot{\gamma }\;\right)}^{m}}$$

The main effects of formate ionic strength on the flow behavior of these solutions are a slight increase in the values of the limiting viscosities (η_o_ and η_∞_) and the consistency index, k, more significant for higher concentrations of potassium formate. Nevertheless, little effect was observed on the values of the flow index, which remains almost constant, around 0.90, with ionic strength.

These observations indicate that for this XG solution, the ionic strength due to potassium formate salts would reinforce the response of the solution in steady-state viscous flow; however, this does not significantly influence the degree of sensitivity to shear. This behavior is compatible with the hypothesis of Wyatt et al. [27] in the sense that the effect of the screening of charge above a certain ionic strength influences interactions that remain independent of the shear rate that flows the solution.

### 3.3. Effect of Aging on the Rheological Behavior

Despite XG in solution having suitable mechanical properties under shear and being considered as a good thickener in many oilfield operations, its resistance to high-temperature environments is a major drawback that limits high-pressure/high-temperature (HP/HT) applicability. The evaluation of the effect that thermal aging exerts on the rheology and microstructure of XG in solution would be a good strategy for determining both the time and applicability window in thermal aggressive environments.

The thermal resistance of XG solutions is enhanced by both the screening protection of charge due to the ionic strength of cations and the antioxidant capacity of the anion [33]. The thermal resistance of XG would improve if the ordered structure remains as the main conformation of the biopolymer in solution at high temperatures [19,21]. In this sense, in previous work, it was proved that brines not only protect XG in solution against thermal disruption, enlarging the temperature interval where the thermal transition takes place, but also that the weak-gel response dominates the rheological properties [9]. However, the original rheological behavior, at temperatures below the thermal transition, was not fully recovered after submitting the solution to high temperatures, even in the presence of concentrate brines. Furthermore, formate salt was the most suitable salt compared with chlorides for protecting the structure of XG in solution against thermal degradation, at concentrations above XG 0.5 wt% [8,20]. These solutions retained the minimum degree of pseudoplasticity to be suitable for engineering applications in aggressive temperature environments, such as drilling and completion operations in oilfields. Recently, these findings have also been corroborated by Wu et al. [21], studying fluids based on XG with side-chain modifications in sodium and calcium concentrate brines at high temperatures.

**Figure 4:**
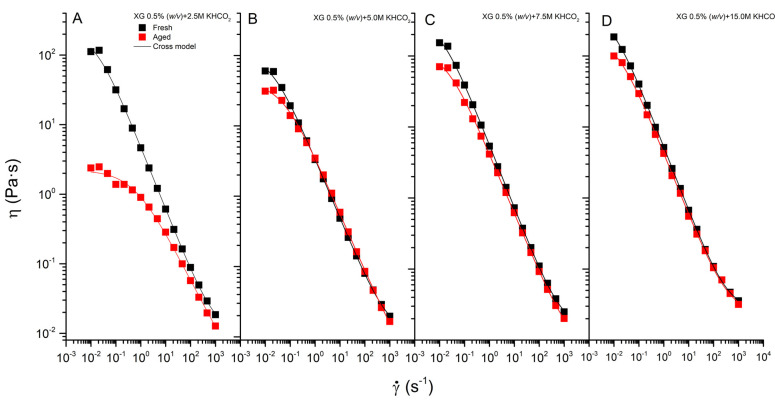
Effect of aging on the flow behavior for XG 0.5% (*w/v*) in potassium formate 2.50 M (**A**), 5.00 M (**B**), 7.50 M (**C**), and 15.00 M (**D**).

Here, XG 0.5% (*w/v*) in potassium formate solutions at concentrations below 1.00 M completely loses its pseudoplastic characteristics after being submitted to static aging for 16 h at 165 °C, resulting in an aged solution that behaves as a low viscosity Newtonian liquid. The residual viscosity of the aged solutions is around 6 mPa·s (see Table 2), slightly above the viscosity of potassium formate, which ranges from 1–8 mPa·s for 0.50 to 15.00 M, respectively. At higher concentrations of salt, as can be seen in Figure 4, the pseudoplasticity is partially recovered at 30 °C. This result suggests that some degree of the ordered structure remains after aging. The flow curves after aging were fitted to the Cross model, with the resulting parameters shown in Table 2.

In general, the higher the concentrate in formate salt, the more resistant it is to degradation; however, some degree of degradation is always observed. To some extent, thermal aging modifies all parameters that characterize the pseudoplastic behavior of XG solutions, as can be deduced by comparing the values of the parameter in Table 1 (before aging) and Table 2 (after aging).

**Figure 5:**
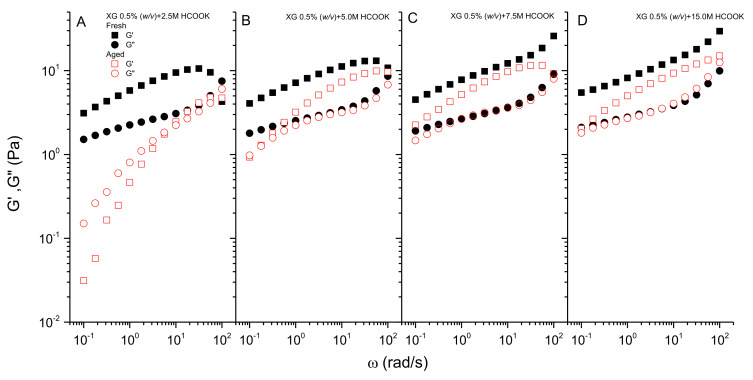
Effect of aging on the viscoelastic behavior for XG 0.5% (*w/v*) in potassium formate 2.50 M (**A**), 5.00 M (**B**), 7.50 M (**C**), and 15.00 M (**D**).

The effect of thermal degradation on the XG structure also changes the viscoelastic characteristic of the solution. In agreement with the previous results of viscous flow tests, XG solutions below the concentration of 1.00 M in potassium formate completely lose their viscoelastic properties, turning into a viscous liquid solution. This result is due to the high degradation of the polymeric structure. XG solution at the concentration of 2.50 M partially retains its original viscoelastic properties, as can be seen in Figure 5A, but after aging, a clear decrease in the values of moduli is noticed. In addition, at low frequencies, the solution shows an apparent tendency to the terminal region of the mechanical spectrum, with values of the viscous modulus higher than those of the storage modulus at the same frequency. In this region, the slope in log scale of the storage and loss moduli is 1.26 and 0.77, relatively close to 2 and 1, respectively. This would indicate that some degree of interaction remains among the degraded biopolymer chains in solution with longer relaxation times. At higher frequencies, a crossover point, at which the loss tangent becomes 1, appears followed by a flattening of the slope of the viscoelastic moduli versus frequency. This fact indicates that part of the weak-gel structure of xanthan remains in solution, becoming a more viscoelastic liquid [12].

The increase in the formate concentration leads to an increase in the values of both moduli, enhancing the viscoelastic properties of the solution. This fact is more evident for the storage modulus, as can be seen in Figure 5B–D, which resists the effects of thermal aging very well. The results indicate less degradation with the increase in the concentration of salt. Consequently, the formate salt would protect against thermal degradation of the ordered structure, which would be responsible for the elastic properties of the solution. Moreover, the crossover point shifts to lower frequencies, extending the frequency windows where the solution displays weak-gel properties. Nevertheless, as is the case of steady-state flow, some degree of degradation is also observed even for concentrate brines. It is worth noting that the percentage of recovery in the values of G_N_^0^ (100·G_N_^0^_Aged_/ G_N_^0^_Fresh_) after aging is 68%, 77%, and 68% for concentrations of 5.00, 7.50, and 15.00 M, respectively.

It is also worth highlighting that in agreement with previous results [15,28], positive deviations of the Cox-Merz rule (steady shear viscosity values lower than those of the complex viscosity at the same shear rate and frequency) have been observed for both fresh and aged XG solutions (i.e., for fresh XG in brine 7.50 M, at 10 s^−1^ or rad/s, η = 0.73 Pa·s, η* = 1.27 Pa·s; for aged XG in brine 7.50 M, at 10 s^−1^ or rad/s, η = 0.62 Pa·s, η* = 1.04 Pa·s). This suggests that the structural degradation due to thermal aging of XG does not change the thermorheological nature in solution [9].

### 3.4. Structure-Properties Relationships

The mechanisms of structural degradation for XG in solution include free-radical oxidation, acid-basic catalyzed hydrolysis, and enzymatic degradation [34,35,36]. It has been stated that the degradation of the structure would start with an attack on the side chains and would continue through the main chain with different effects on the rheological behavior [18]. Side-chain degradation would mainly affect the acetyl group, with there being less influence on the reduction of the elastic properties of the solution since the main ordered structure remains [21]. Further attacks to the main chain would drastically change the molecular weight and the rheological properties.

In this study, the changes in pH and the products of degradation were determined to address the changes in XG structure. The pH drop after high-temperature aging has been associated with both free-radical oxidation [35,37] and acid-basic hydrolysis [36,38]. The concentration of F+HMF in the aged solution would be a measure of the degradation of the main chain. At high temperatures, the degradation of hexoses and pentoses by acid-hydrolysis converts them into F+HMF, respectively. These compounds can be determined using UV-vis spectroscopy in polysaccharide solutions after aging. This has been related to chain degradation even in the presence of formate salt [26].

At 165 °C, the disordered conformation would be the main structure for XG in potassium formate solution at concentrations below 5.00 M [39]. For these solutions, XG in a disordered conformation would be very vulnerable to hydrolysis of the main chain [18,19], resulting in the loss of the rheological properties after 16 h of exposure at 165 °C in a low-oxygen environment. For these solutions, the ionic strength is not sufficient to raise the order-disorder transition temperature above the aging temperature and prevent XG chains from chemical attack [21].

**Figure 6:**
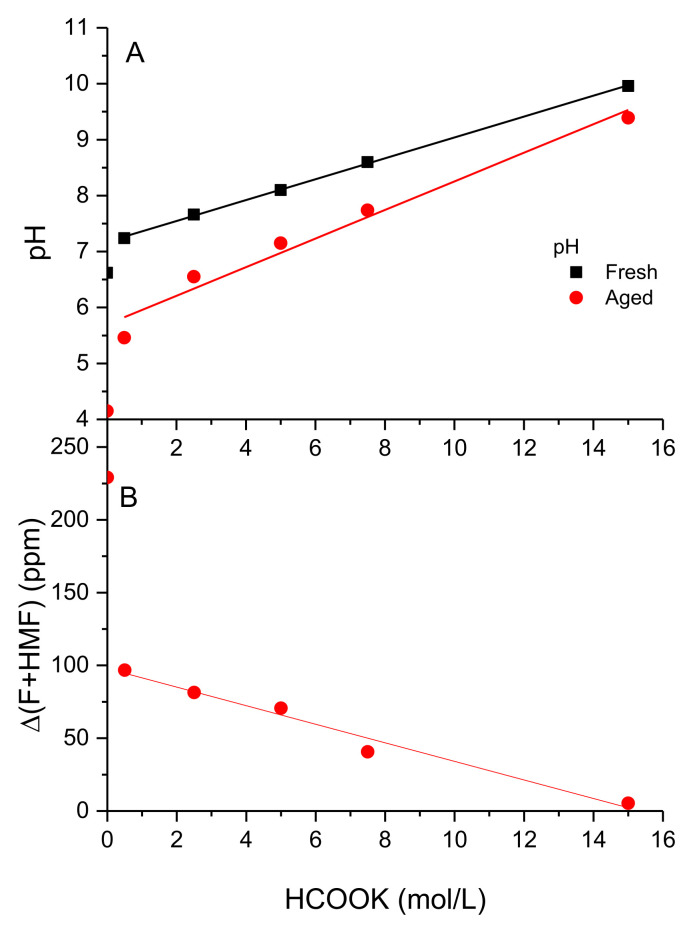
Degradation measured as: (**A**) Evolution of pH. (**B**) Furfural and hydroxymethyl/furfural content.

In this regard, the evolution of pH and the increase in F+HMF content of the solutions after degradation are displayed in Figure 6A,B, respectively. As can be seen in Figure 6A, aging leads to a decrease in pH inversely proportional to the formate content. Native XG solution shows acidic pH and, consequently, the highest degree of degradation due to acid-hydrolysis would be expected. In addition, the highest content in F+HMF would also indicate the degradation of the main chain of XG, and consequently, the loss of the rheological properties. The concentration in potassium formate increases the pH of both fresh (R^2^ = 0.99) and aged (R^2^ = 0.96) solutions linearly up to 9.9 and 9.3 for the saturated solution, respectively. Degradation due to thermal aging acidifies the resulting solution; however, the pH drop is flattened when the concentration of potassium formate increases [26]. This observation would be explained assuming the high protection capacity of the ordered structure by formate salt [37] and subsequently the delay in degradation processes for concentrations higher than 5.00 M, where the ordered state remains at aging temperature. Since fresh samples were pressurized with N_2_ at 50 bar, free radical oxidation would be minimized and, therefore, degradation by hydrolysis seems to be the most probable mechanism, showing a different extent depending on the formate concentration.

However, the content in F+HMF decreases suddenly with the presence of formate 0.50 M (from 235 to 100 ppm), and evolutes inversely and proportionally to the formate content with a slope of −6.9 M (R^2^ = 0.97), being 5 ppm for 15.00 M. A high degree of resistance to thermal degradation with important maintenance of both pseudoplasticity and elastic properties (Figure 4 and Figure 5, respectively) is observed for aged solutions in HCOOK above 7.50 M. These solutions show a (F+HMF) content below 50 ppm. Therefore, XG in potassium formate above 7.50 M (equivalent to 48.2 wt% in HCOOK and density at 30 °C of 1.310 g/mL) shows rheological behavior suitable for use in oilfield applications. It is worth noting that the density of the solution does not change after aging at 165 °C for 16 h, being very similar to that of the fresh solution for all concentrations of formate tested.

## 4. Conclusions

In linear viscoelasticity, XG at 0.5% (*w/v*) behaves as a weak gel, with values of the storage modulus being higher than those of the loss modulus at the same frequency. The gel strength increases with the concentration of formate in solution. The main effects on the flow behavior of the formate ionic strength are a slight increase in the values of the limiting viscosities, (η_o_ and η_∞_) and the consistency index, k.

In general, the higher the concentrate in formate salt, the more resistant it is to degradation after aging. Aged XG solutions in formate below 1.00 M completely lose both their elasticity and their pseudoplasticity, resulting in a low-viscosity Newtonian liquid. XG in formate solutions below 5.00 M would develop a disordered structure that is very vulnerable to the hydrolysis attack of the main chain, resulting in a loss of rheological properties after 16 h at 165 °C.

At higher formate concentrations, XG retains its ordered structure responsible for the high rheological properties. This structure would be protected by high ionic strength against the thermal degradation of aging at 165 °C; however, some degree of degradation always takes place, lowering all the parameters that characterize the pseudoplastic behavior and the values of the storage modulus. Nevertheless, solutions above 7.50 M in formate retain the rheological properties enough to be suitable for use in oilfield applications.

## Figures and Tables

**Table 1:** Cross model parameters for fresh samples XG 0.5% (*w/v*) as a function of the formate concentration.

Fresh Xanthan 0.5% (*w/v*)				
KHCO_2_ (mol/L)	η_0_ (Pa·s)	η_∞_ (Pa·s)	k (s)	m
0	145.98	6.02 × 10^−3^	52.3	0.881
0.25	186.06	7.39 × 10^−3^	61.5	0.894
0.5	221.43	7.47 × 10^−3^	61.9	0.890
1	191.68	7.82 × 10^−3^	63.1	0.891
2.5	216.60	9.34 × 10^−3^	68.9	0.899
5	266.08	1.14 × 10^−2^	73.7	0.907
7.5	328.67	1.40 × 10^−2^	94.6	0.893
15	428.43	2.63 × 10^−2^	129.7	0.904

**Table 2:** Cross model parameters for aged samples XG 0.5% (*w/v*) as a function of the formate concentration.

HCOOK (mol/L)	η_0_ (Pa·s)	η_∞_ (Pa·s)	k (s)	m	η_0Ag_/η_0Fr_ (%)	k_Ag_/k_Fr_ (%)	m_Ag_/m_Fr_ (%)
0	6.5 × 10^−3^		-	-			
0.25	6.4 × 10^−3^		-	-			
0.50	6.7 × 10^−3^		-	-			
1.00	7.8 × 10^−3^		-	-			
2.50	2.200	1.00 × 10^−3^	1.70	0.707	1.00	2.40	78.6
5.00	41.41	2.64 × 10^−3^	19.3	0.817	15.2	26.1	90.1
7.50	120.60	7.50 × 10^−3^	52.3	0.847	36.7	55.3	94.8
15.00	192.00	2.39 × 10^−2^	77.1	0.874	44.8	59.4	96.7

## Data Availability

The raw/processed data required to reproduce these findings cannot be shared at this time due to technical or time limitations.
